# Proteomic Analysis of Aqueous Humor Identified Clinically Relevant Molecular Targets for Neovascular Complications in Diabetic Retinopathy

**DOI:** 10.1016/j.mcpro.2025.100953

**Published:** 2025-03-19

**Authors:** Jae Won Oh, Seong Joon Ahn, Jae Hun Jung, Tae Wan Kim, Kwang Pyo Kim

**Affiliations:** 1 Department of Applied Chemistry, Institute of Natural Science, Global Center for Pharmaceutical Ingredient Materials, Kyung Hee University, Yongin, Republic of Korea; 2 Department of Biomedical Science and Technology, Kyung Hee Medical Science Research Institute, Kyung Hee University, Seoul, Republic of Korea; 3 Department of Ophthalmology, Hanyang University Hospital, Hanyang University College of Medicine, Seoul, Korea; 4 Department of Ophthalmology, SNU Blue Eye Clinic, Seoul, Korea

**Keywords:** mass spectrometry, diabetic retinopathy, aqueous humor, proteomics, clinical biomarker

## Abstract

Diabetic retinopathy (DR) is a leading cause of blindness in adults under 40 in the developed world, with a significant proportion progressing to vision-threatening stages such as proliferative diabetic retinopathy (PDR) and neovascular glaucoma (NVG). This study aims to explore the molecular mechanisms underlying the progression from nonproliferative DR to PDR and NVG, focusing on identifying potential biomarkers and therapeutic targets. Utilizing discovery-based proteomics, specifically label-free quantification and tandem mass tag, we analyzed aqueous humor (AH) proteins obtained during cataract surgery or anterior chamber paracentesis from patients with nonproliferative DR, PDR, and NVG. Validation of marker candidates for each disease state was conducted using triple quadrupole-MS for targeted protein quantification. Our proteomic analysis identified 2255 proteins, and gene ontology analysis and functional annotation highlighted key biological processes implicated in DR, such as lens development, immune responses, and lipid metabolism. Validation of potential biomarkers identified 20 proteins with significant concentration changes, including several candidates with diagnostic utility based on ROC curve analysis. Further investigation into clinical relevance revealed that crystallin gamma-S is strongly associated with cataract severity, highlighting its role as a potential marker for ocular complications in DR. Importantly, we identified that the pathological factors driving DR progression have a much greater impact than age, a previously known variable, in shaping the proteomic landscape of AH. Additionally, proteins associated with macular degeneration (CA1, CA2, and HBA1) were uncovered, providing new insights into overlapping mechanisms between DR and other retinal diseases. Finally, proteins linked to panretinal photocoagulation treatment, including APOB and CST6, were identified, suggesting their involvement in the therapeutic response and post-treatment adaptation. These findings underscore the potential of AH proteomics in uncovering predictive biomarkers and elucidating the molecular pathogenesis of DR and its complications.

## Highlights

• Proteomic profiling of AH in DR patients identified a total of 2,255 distinct proteins.
• Key biological processes implicated in DR include lens development, immune responses, and lipid metabolism.
• Crystallin gamma-S (CRYGS) was strongly associated with cataract severity, serving as a potential marker.
• Age was found to have minimal impact compared to pathological factors in shaping the AH proteomic landscape.
• Proteins linked to macular degeneration (CA1, CA2, HBA1) and PRP treatment (APOB, CST6) were identified.

## In Brief

Using quantitative proteomics, this study characterizes molecular alterations in aqueous humor across diabetic retinopathy stages, from NPDR to NVG. Biomarker candidates associated with disease progression, cataract severity, macular degeneration, and treatment response were identified and validated. Clinically relevant molecules offer insights into the molecular mechanisms of diabetic ocular complications, highlighting the utility of AH proteomics for translational biomarker discovery.

## Graphical Abstract

**Figure:**
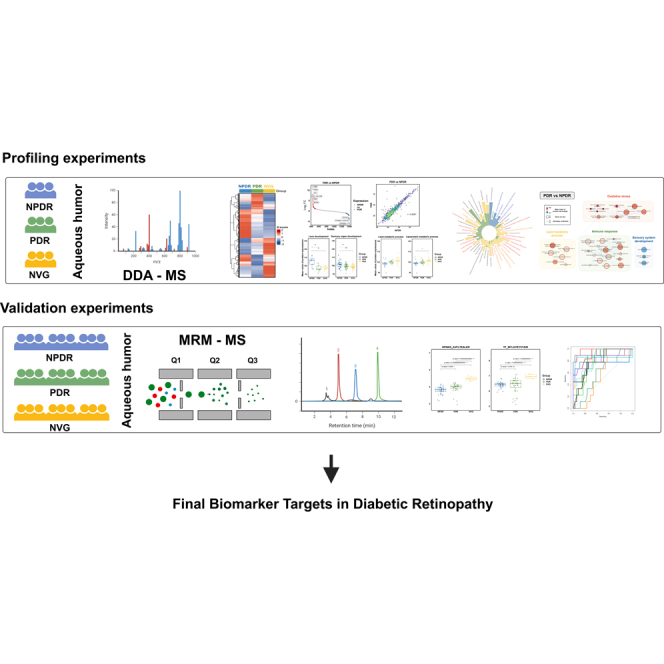

Diabetic retinopathy (DR) remains the leading cause of blindness and vision loss among adults aged under 40 years in the developed world (1, 2). Previous studies have shown that approximately one-third of diabetic patients exhibit signs of DR, with approximately one-10th reaching vision-threatening stages such as proliferative diabetic retinopathy (PDR) (3, 4). PDR, an advanced stage of DR characterized by neovascularization, is responsible for a significant number of cases of diabetes-related blindness, with approximately 8000 eyes affected annually in the United States alone (5).

DR develops as a result of microvascular retinal changes induced by hyperglycemia (6). Prolonged exposure to elevated blood sugar levels leads to structural and functional alterations in retinal blood vessels, including thickening of the basement membrane, pericyte loss, endothelial cell dysfunction, and capillary occlusion (7, 8, 9). These changes result in impaired blood flow, ischemia, and hypoxia within the retina. Ischemic areas of the retina release angiogenic factors, such as vascular endothelial growth factor, which stimulate the formation of new blood vessels through angiogenesis, contributing to the development of PDR.

The abnormal growth of neovascular tissue also affects the eye's drainage system, causing impaired outflow of aqueous humor (AH) (10, 11). Neovascular glaucoma (NVG), a specific subtype of glaucoma, arises due to neovascular complications associated with PDR. Invasion of the anterior chamber angle by these new vessels obstructs the trabecular meshwork, the primary drainage pathway for AH. This increased resistance to outflow leads to elevated intraocular pressure (IOP), a hallmark of NVG. While the causal relationship between diabetes and glaucoma remains a topic of ongoing debate, NVG has a well-established link to neovascularization and diabetic complications (11, 12). Delayed diagnosis or inadequate management at this stage can result in complete vision loss or even loss of the eye itself (10, 11). Hence, investigating the molecules associated with the progression from nonproliferative diabetic retinopathy (NPDR) to PDR and NVG is crucial for preventing blindness and identifying potential therapeutic targets. However, the specific mediating molecules, molecular processes, and diagnostic biomarkers related to NPDR, PDR, and NVG are not yet fully understood.

Proteomics offers a powerful tool for analyzing complex protein mixtures and identifying biomarkers (13, 14). Among the various methods available, mass spectrometry (MS)-based proteomics has proven to be particularly effective in large-scale protein identification and quantitation (13). This technique has been applied to profile proteins in body fluids, including plasma, vitreous humor, and AH (15, 16, 17). Characterizing the AH proteome can provide valuable insights into the pathogenesis of serious complications in DR and lay the foundation for biomarker discovery (18). Importantly, AH can be easily obtained during cataract surgery or anterior chamber paracentesis for controlling increased intraocular pressure, with minimal additional risk to the patient (19).

In this study, we employed discovery-based proteomics to analyze AH proteins obtained from patients with NPDR, PDR, and NVG who underwent cataract surgery or anterior chamber paracentesis for therapeutic purposes. We validated marker proteins for each disease state using triple quadrupole-MS, a specialized technique for targeted protein quantification in complex samples. We aimed to identify a panel of AH proteins that exhibit differential expression between NPDR and PDR, as well as between PDR and NVG, to investigate biomarkers and shed light on the pathogenesis of these serious neovascular complications.

## Experimental Procedures

### Patient and Controls

Patients and controls were recruited from the retina clinic at the Department of Ophthalmology of Seoul Metropolitan Government Seoul National University Boramae Medical Center between July 2013 and February 2017. This study was conducted in accordance with the principles outlined in the Declaration of Helsinki and was approved by the institutional review board [IRB No. 16-2017-4]. Informed consent was obtained from all participants involved in the study. Human biological materials were handled with strict confidentiality measures, including the de-identification and coding of biospecimens to protect participant privacy. Approval was obtained from the Investigational Review Board of SMG-SNU BMC Clinical Research Institute, and all subjects provided written informed consent after receiving a detailed explanation of the research purpose.

Patients with other etiologies such as retinal vein occlusion and ocular ischemic syndrome were excluded after a comprehensive ophthalmic examination, which included slit-lamp biomicroscopy, indirect ophthalmoscopy, fundus photography, fluorescein angiography, and spectral domain optical coherence tomography. Patients who had previously received intravitreal injections or intraocular surgery or recent (within 3 months) panretinal photocoagulation at the time of enrollment were also excluded from the study.

### Experimental Design and Statistical Rationale

Our study included 47 patients with NPDR who underwent cataract surgery, 48 patients with PDR undergoing vitrectomy for the treatment of vitreous hemorrhage or tractional retinal detachment, and 17 patients with NVG undergoing Ahmed implant surgery for uncontrolled intraocular pressure. AH samples were obtained during the surgical procedures through anterior chamber paracentesis. These samples were divided into two independent cohorts to profile and validate the selected biomarker candidate proteins: a profiling cohort (10 patients with NPDR, 10 patients with PDR, and eight patients with NVG) and a validation cohort (37 patients with NPDR, 38 patients with PDR, and nine patients with NVG). The sample sizes for the validation cohort were determined using *a priori* power analysis based on the effect size (d = 0.759) calculated from the profiling analysis. To achieve a statistical power of 90% (1-β = 0.9) at a high significance level (α = 0.05), a total of 73 participants (approximately 37 per group) was required. The sample size was calculated using the formula:

$$n=\frac{{\left(Z_{a}+Z_{b}\right)}^{2}*2}{d^{2}}$$

Where Z_a_ (1.96 for α = 0.05) and Z_b_ (1.28 for 1-β = 0.9) are the critical values for the significance level and statistical power, respectively. While the NPDR (n = 37) and PDR (n = 38) groups met this requirement, the NVG group (n = 9) reflects practical limitations due to the rarity of this condition. Despite this, all available NVG samples were included to ensure statistical robustness and maximize representation. Due to the low abundance of proteins in AH and the limited volume obtainable from human subjects, depleting high-abundant proteins significantly reduces the protein yield, complicating comprehensive protein analysis. To address this in profiling experiments, profiling cohorts at each stage were pooled to ensure sufficient protein quantities for the profiling study. For validation study, every validation patient sample was analyzed in duplicate (two technical replication). There were no significant differences in age, sex, or hypertension among the patient groups in the profiling and validation cohorts (Table 1). Additional clinical information is in supplemental Table S1. In the absence of AH samples from normal individuals, NPDR, as the least progressive stage, was designated as the control group in the statistical analysis.

**Table 1:** Clinical characteristics of patients in the nonproliferative diabetic retinopathy (NPDR), proliferative diabetic retinopathy (PDR), and neovascular glaucoma (NVG) groups within the profiling and validation sets

Characteristics	Profiling set					Validation set					*p* (profile *versus* validation)
	Total	NPDR	PDR	NVG	*p*^a^	Total	NPDR	PDR	NVG	*p*^a^	
Age, years (range)	61.6 ± 12.1 (38–82)	66.2 ± 11.0 (50–82)	62.3 ± 11.9 (38–79)	54.9 ± 11.8 (38–77)	0.137	59.8 ± 10.6 (30–83)	66.1 ± 8.3 (44–83)	54.9 ± 8.9 (31–78)	53.3 ± 12.1 (30–68)	<0.001	0.700
Sex, male: female	16 (57.1%): 12 (42.9%)	5 (50%): 5 (50%)	4 (40%): 6 (60%)	7 (87.5): 1 (12.5%)	0.154	46 (54.8%): 38 (45.2%)	18 (48.6%): 19 (51.4%)	23 (60.5%): 15 (39.5%)	5 (55.6%): 4 (44.4%)	0.600	0.826
Hypertension	14 (50%)	4 (40%)	6 (60%)	4 (50%)	0.889	47 (56.0%)	21 (56.8%)	21 (55.3%)	5 (55.6%)	1.0	0.584
Combined ophthalmic diseases	0	0	0	0	1.0	2^a^ (2.4%)	2^a^ (5.4%)	0	0	0.395	1.0

a epiretinal membrane in two cases.

### Proteomic Analysis of AH Samples

**Fig. 1:**
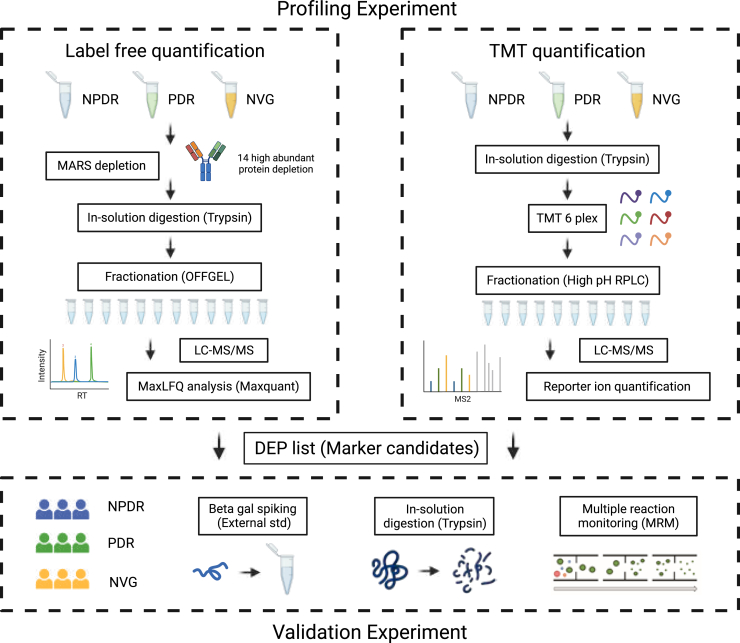
**Overall proteomics workflow.** Schematic diagram depicting overall experimental workflow of the proteomics experiments. AH samples were lysed and digested into peptides. Digested peptides were prefractionated with OFFGEL fractionation and High pH RPLC in order to identify more proteins. With LC-MS/MS, fractionated peptides were analyzed, and LFQ and TMT method was adopted for data analysis. AH, aqueous humor; LC, liquid chromatography; LFQ, label-free quantification; TMT, tandem mass tag.

AH samples were obtained by anterior chamber paracentesis as the initial step of planned intraocular surgery. The samples were collected in sterile Eppendorf tubes and rapidly frozen at −80 °C until further analysis. Due to the exceptionally low protein concentration (0.1 ∼ 0.3ug/ul) in AH and the limited volume (5–20 μl) obtainable from each patient, conducting a comprehensive proteomics analysis with depletion of high abundant proteins requires pooling samples. For this study, we collected 10 μl of AH from each group patients, pooling the samples to obtain ∼100 μg of total protein, which was subsequently used for profiling experiments. To create profiling pools for each group, individual samples from each group (NPDR: 10, PDR: 10, NVG: 8) were pooled respectively in equal amounts. Pooled samples were used for label-free quantification (LFQ) analysis (20, 21) and tandem mass tag (TMT) relative quantification analysis in order to quantify overall AH protein expression and find out statistical significant protein candidates. The candidate targets were later validated through individual sample (NPDR: 37, PDR: 38, NVG: 9) to obtain statistical significance using multiple reaction monitoring (MRM). Figure 1 provides a flow diagram summarizing our experimental strategy for quantitative comparisons of AH proteins in patients with NPDR, PDR, and NVG.

### Profiling Experiments of DR in AH

AH samples pooled for the profiling experiment underwent the depletion of high-abundance proteins, including albumin, IgG, antitrypsin, IgA, transferrin, and haptoglobin, using the multiple affinity removal system (MARS) −14 spin cartridge (Agilent) in order to identify low abundant proteins that cannot be detected without removal of high abundant proteins. Only samples for LFQ were depleted, and nondepleted samples were digested with in-solution digestion method for TMT pipeline. Nondepleted peptides were TMT labeled, pooled, and fractionated in to 10 fractions using high-pH reversed phase fractionation. Depleted AH proteins were digested with in-solution digestion method and fractionated into 12 fractions using isoelectric point-based OFFGEL fractionation. After fractionation, the fractions were harvested, dried using a speed-vac, and subjected to clean-up using C18 spin column (Thermo Fisher Scientific). Desalted peptides were resuspended in 0.1% formic acid (FA) and analyzed using the Q-Exactive Orbitrap hybrid mass spectrometer (Thermo Fisher Scientific) coupled with an EASY-nLC 1000 system (Thermo Fisher Scientific). Peptides were eluted through a trap column, ionized *via* an Easy spray equipped with a 50 cm × 75 μm ID column packed with 2 μm C18 particles, and subjected to the Q-Exactive Orbitrap mass spectrometer for the acquisition of tandem mass spectrometric data. Raw files for LFQ were processed using the open-source software MaxQuant (v. 1.5.1.2) for LFQ, and a cutoff of 1% was applied at both the peptide and protein levels. Raw files for TMT were processed using Proteome discoverer (ver 3.0), and strict 1% false discovery rate (FDR) was applied on both protein and peptide levels. Normalized LFQ intensity and reporter ion intensity were used for further statistical analysis, and differentially expressed proteins (DEPs) were enriched. Gene ontology analysis and functional annotation and network analysis was held for understanding the DR process.

### Multiple Affinity Depletion of Abundant Proteins Using MARS

AH samples pooled for protein profiling underwent the depletion of high-abundance proteins, including albumin, IgG, antitrypsin, IgA, transferrin, and haptoglobin, using MARS −14 spin cartridge (Agilent). First, particulates were removed by centrifuging the sample (100 μg) at 16,000 g for 1.5 min. The flow-through was then mixed with buffer A (Agilent) for depletion. During depletion, the flow-through was collected up to 600 μl, and the protein concentration was determined using a bicinchoninic acid assay.

### In-Solution Digestion

The in-solution digestion was performed using the following method: The depleted sample was mixed with 8M urea in 100 mM ABC (ammonium bicarbonate), followed by incubation at room temperature for 20 min. In the TMT pipeline, rather than using ABC, triethylammonium bicarbonate was used for avoiding poor labeling of TMT chemicals. Vortexing and sonication were performed three times during the incubation period. Next, 10 mM DTT was added to the sample for reduction, and the reaction occurred at room temperature for 1 h. Subsequently, 30 mM IAA was added for alkylation, and the sample was kept in the dark for 30 min to complete the reaction. After alkylation, the sample was diluted with 100 mM ABC to bring the urea concentration below 1M. Trypsin was then added to the sample at a ratio of 1:50 (trypsin:sample) and incubated overnight at 37 °C. Any remaining activated trypsin was acidified using FA, and the peptides were desalted using a c18 Harvard macro spin column.

### OFFGEL Fractionation

Isoelectric point-based OFFGEL fractionation for peptides was carried out according to the manufacturer's instructions. Peptides were fractionated into 12 fractions using the 3100 OFFGEL fractionator (Agilent Technologies). Dried peptides were reconstituted with isoelectric focusing buffer (GE Healthcare) containing 5% glycerol and 1% ampholytes. Each well containing immobilized pH gradient strips (Immobiline Drystrip pH 3-10 NL, 24 cm, GE Healthcare) was rehydrated with 40 μl of buffer for 20 min, and then 150 μl of isoelectric focusing buffer with peptides was added per well. Filter papers soaked in buffer were placed at the ends of the gel strips, and mineral oil was overlaid to prevent drying. Isoelectric focusing was conducted at 4,500V with a limiting current of 50 μA and a focusing target of 50 kVh at 20 °C overnight. After fractionation, the 12 fractions were harvested, dried using a speed-vac, and subjected to cleanup using C18 spin column (Thermo Fisher Scientific).

### TMT Labeling and Fractionation

Digested peptides were labeled with TMT isobaric tags (Thermo Fisher Scientific) according to the manufacturer’s protocol. Peptides from the NPDR group were labeled with TMT channels 126 and 129, PDR group with 127 and 130, and NVG group with 128 and 131. After labeling, the samples were pooled together to ensure equal representation of all groups.

The pooled samples were fractionated into 10 fractions using an Accucore 150 C18 LC column (150 × 2.1 mm, 4 μm particle size) on an Agilent 1200 series high-performance liquid chromatography system. Fractionation was performed under alkaline conditions using the following gradient: 0 to 10 min, 5% mobile phase B; 10 to 70 min, 35% mobile phase B; 70 to 80 min, 70% mobile phase B; and 80 to 105 min, 5% mobile phase B. Mobile phase A consisted of 10 mM ammonium formate (pH 10), and mobile phase B consisted of 10 mM ammonium formate with 90% acetonitrile (pH 10).

Fractions were collected, concentrated, and subsequently desalted using a C18 spin column following standard protocols to remove any remaining impurities. The desalted peptides were dried under vacuum and stored at −80 °C until further use in downstream proteomic analysis.

### Protein Identification by LC-MS/MS

The desalted fractionated peptides were resuspended in 0.1% FA and analyzed using the Q-Exactive Orbitrap hybrid mass spectrometer (Thermo Fisher Scientific) coupled with an EASY-nLC 1000 system (Thermo Fisher Scientific). For proteome profiling analysis, a linear gradient was employed, starting from 5% to 35% of solvent B over 180 min, followed by a gradient from 35% to 80% of solvent B in 5 min. The column was then held at 80% of solvent B for 20 min, followed by equilibration at 1% for 45 min. Peptides were eluted through a trap column, ionized *via* an Easy spray equipped with a 50 cm × 75 μm ID column packed with 2 μm C18 particles, and subjected to the Q-Exactive Orbitrap mass spectrometer for the acquisition of tandem mass spectrometric data.

MS scans were acquired in the range of 400 to 2000 Th at a resolution of 70,000 (at m/z 400) using an automated gain control target value of 1.0 × 10^6^ and a maximum ion injection time of 120 ms. The 10 most abundant ions with a charge of ≥ 2 to 6 were dynamically selected with an isolation width of 2 Th and fragmented by higher energy collisional dissociation with a normalized collision energy of 27. The maximum ion injection time for MS/MS was set to 60 ms at a resolution of 17,500. Dynamic exclusion time was set to 30 s. For the TMT pipeline, the fixed first mass was set to 100, and the normalized collision energy was set to 30 during MS acquisition.

### Raw Data Processing

All LFQ raw files were processed using the open-source software MaxQuant (v. 1.5.1.2) and TMT raw files were processed using Proteome Discoverer (v3.0) with uniprot human database (229,959 entries, Swissprot and trembl database released in 2023.09) (8). The MS2 spectra were searched using the Andromeda search engine for LFQ data, Sequest HT for TMT data with strict trypsin specificity that required cleavage C-terminal after K or R, allowing for up to two missed cleavages. Carbamidomethylation of cysteine was considered a fixed modification, while oxidation of methionine was considered variable modifications for the search. In the case of TMT, TMT 6plex modifications for lysine and N-term were considered as fixed modifications. The minimum required peptide length was set to six amino acids. To control the FDR, a cutoff of 1% was applied at both the peptide and protein levels. A maximum initial precursor mass tolerance of up to 20 ppm and a fragment mass tolerance of up to 0.6 Da were allowed. Protein identification required at least one razor + unique peptide for confident identification. The extracted ion chromatogram-based LFQ algorithm in MaxQuant and reporter ion intensity extraction were used to quantify the proteins. For data processing in MaxQuant, the "match between runs" option was utilized for nonlinear retention time alignment, with a match time window of 0.7 min and an alignment time window of 20 min.

### Data Analysis of Profiling Experiments

Each LFQ intensity for NPDR, PDR, and NVG was transformed into log2 values. Statistical quantification analysis of the log-transformed data was performed using a two-sample *t* test (*p*-value <0.05). In cases where missing values were present, relative quantification of proteins was performed by comparing the expression of proteins in one group with missing values to the average expression of the other groups with no missing values. Quantitative proteomic data from TMT and LFQ pipelines were integrated using a normalization strategy to ensure comparability across platforms. Batch effect between the LFQ and TMT were corrected using Combat in sva packages. The integrated dataset was then subjected to non-negative matrix factorization (NMF) analysis to identify patterns of protein expression and cluster samples based on shared molecular features.

For the clinical data, descriptive statistics were used. Odds ratios were calculated to evaluate the association between DR and clinical characteristics. AH concentrations of DEPs were compared between patients with NPDR and PDR, as well as between those with PDR and NVG, using the fold change of protein expression and Student's *t* test. Statistical significance was defined as *p* < 0.05. Continuous values are presented as mean ± SD. All statistical analyses were performed using R software (version 4.0.1).

### Enrichment Analysis of GO and Network Analysis

To explore cellular processes, pathways, and subcellular localization represented by DEPs, we identified gene ontology (GO) biological process, KEGG pathway, and Reactome with adjusted *p*-values computed from gene set enrichment analysis less than 0.05. To construct a network describing enriched cellular processes, we selected DEPs that are involved in enriched cellular processes. We then built a protein network model using the interaction information of the proteins obtained from KEGGviewer and Enrichment-map using Cytoscape (version 3.10).

### Validation of Selected Candidate Proteins Obtained by Quantitative Analysis Using MRM

For validation of novel biomarkers in the current study, 84 samples, including 38 with NPDR, 37 with PDR, and 9 with NVG were collected. Target peptides of candidate proteins from profiling experiment were designed from SRMatlas with strict threshold. Tryptic peptides with no missed cleavages, which can represent a particular protein, with a length between 6 and 30 amino acids, and with no cysteine or methionine residues to avoid possible modifications. Two or more unique peptides were selected for representing a protein, and three transitions (y and b ions) per a peptide were designed for finding a perfect match from transition to protein. Since the contaminant ions, not peptides, can be included in the window of fragmentation, we selected only product ions whose m/z value are larger than that of the precursor in the notion that the m/z value of product ion from more than 2+ ions can be larger than exact mass of precursor ion.

Each sample (50ug) was digested into peptides using in-solution digestion with trypsin. For the quality check among the samples, beta-galactosidase peptides from *Escherichia coli* (APLDNDIGVSEATR, VDEDQPFPAVPK, IDPNAWVER, VNWLGLGPQENYPDR) were spiked in to sample before digestion. Digested peptides were desalted with C18 spin-column and analyzed with the Agilent 6490 triple-quadrupole mass spectrometer coupled with Agilent LC 1650. RP-high-performance liquid chromatography column (150 × 2.1 mm ID, Agilent Zorbax Eclipse Plus C18 Rapid Resolution HD, 1.8 μm particles) were used for separating peptides. Multi-step LC gradient [from 5% to 45% of solvent B (0.1% FA in 90% ACN) in 25 min, from 45% to 80% of solvent B in 2 min, and holding at 80% of solvent B with 2 min and equilibrate column at 5% in 1 min] with a flow rate of 0.25 ml/min over 30 min at 40 °C was adopted. Dynamic MRM mode with a minimum dwell time of 20 ms and cycle time of 1000 ms was used in order to analyze several candidate peptides in 1 MS run per sample and get at least 10 data points per transitions. Each sample has an experimental duplicate. MS data were pre-processed in skyline (version 22.2.0.312) and the precursor and product ion information. Only the peptides that have more than three transitions considered as identified peptide and quantified when its peak area is exceeding 10ˆ3. Only the peptides those have less than 20% coefficient variance passed for further analysis. Peak area from each peptide were extracted and normalized using the beta-galactosidase peptides and calculated into fold change and *p*-values from Wilcoxon Rank-Sum Test. Statistical significance was defined as *p* < 0.05. The predictability of novel AH biomarkers for PDR or NVG was assessed by receiver operating characteristic curve analysis. Regression analysis was performed to assess the association between protein expression levels and clinical characteristics, including treatment history and disease stages. To address potential batch effects in the proteomic data, batch correction was performed using the Combat algorithm from the sva package in R. Age, DR groups are normalized to check the effect of other clinical variables. This ensured that technical variability across batches was minimized, allowing for more accurate comparisons between groups. All statistical analyses were performed using R software (version 4.0.1).

## Results

### Demographic and Clinical Characteristics

**Fig. 2:**
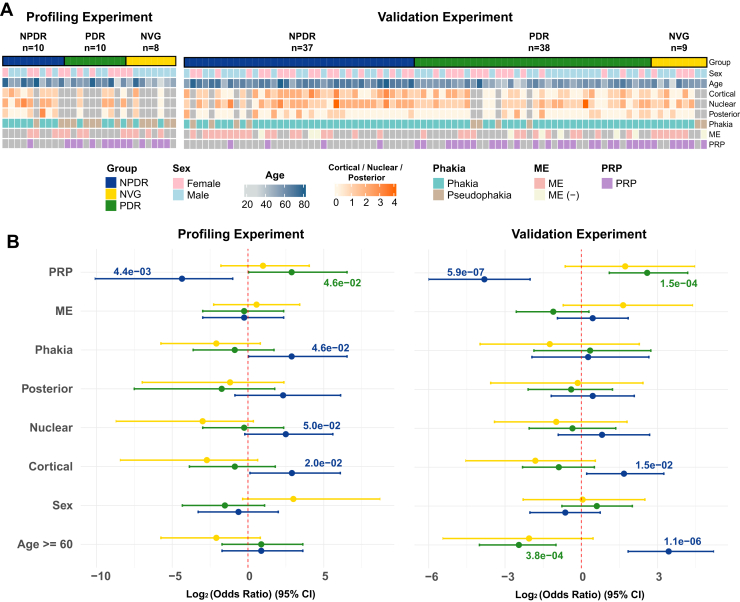
**Clinical characteristics and odds ratio analysis of diabetic retinopathy patients in profiling and validation experiments.***A*, distribution of clinical variables, including sex, age, cataract grades (cortical, nuclear, posterior), phakia/pseudophakia status, macular edema (ME), and panretinal photocoagulation (PRP), across NPDR, PDR, and NVG groups in both profiling (n = 28) and validation (n = 84) experiments. Differences in the distribution highlight group-specific clinical characteristics. *B*, odds ratio analysis for significant clinical variables, showing the relationship between patient characteristics (age ≥60, sex, cataract grades, ME, and PRP) and the likelihood of progression across DR groups. Log2-transformed odds ratios with 95% confidence intervals are presented for both profiling and validation experiments, identifying key clinical factors associated with disease progression.

The demographic characteristics of the enrolled patients with DR are summarized in Table 1 and supplemental Table S1. The demographic and clinical characteristics of the NPDR, PDR, and NVG groups were analyzed in both profiling and validation experiments (Fig. 2). Significant differences were observed in age, sex distribution, cataract grades (cortical, nuclear, posterior), phakia/pseudophakia status, macular edema (ME), and panretinal photocoagulation (PRP) treatment among the groups. Odds ratio analysis further highlighted the strong associations between PRP treatment, and cataract grades with disease progression, underscoring the influence of these clinical factors on group stratification. The mean age of the patients in the pooling and validation sets was 61.6 ± 12.1 years and 59.8 ± 10.6 years, respectively. In the pooling and validation groups, there were 16 (57.1%) and 46 (54.8%) men, respectively. No significant differences were observed in age, sex, hypertension, and combined ophthalmic diseases between the pooling and validation sets (all *p* > 0.05).

### Profiling Experiments of DR in AH

**Fig. 3:**
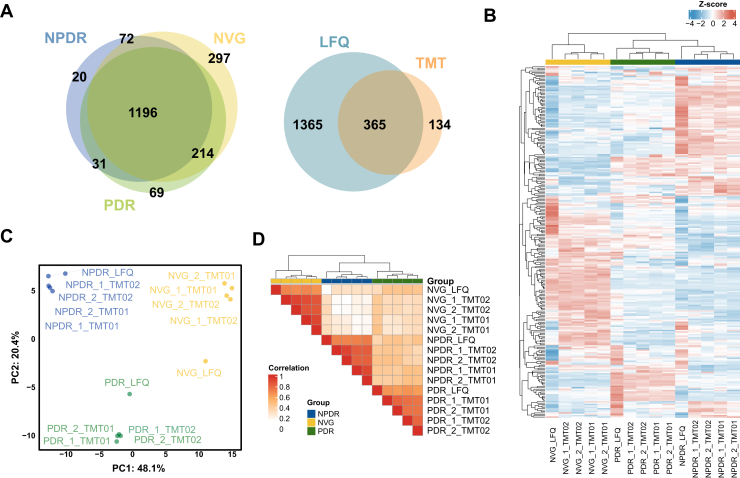
**Integration of TMT and LFQ platforms with clustering and correlation analysis.***A*, Venn diagram showing protein overlap across NPDR, PDR, and NVG groups identified by TMT and LFQ. A total of 365 proteins were commonly identified, while 1365 were unique to LFQ and 134 were unique to TMT. *B*, Z-score normalized heatmap of significant proteins across NPDR, PDR, and NVG groups, revealing distinct clustering patterns between groups. *C*, principal component analysis (PCA) plot of samples, highlighting clear group segregation based on TMT and LFQ proteomic data. *D*, correlation heatmap showing high Pearson correlations among replicates within groups, validating consistency across technical replicates and platforms.

Proteomic analysis identified a total of 2255 proteins in the AH samples. Using the LFQ platform, 1,319, 1,510, and 1779 proteins were identified in patients with NPDR, PDR, and NVG, respectively. A Venn diagram illustrated that 1196 proteins were commonly identified in all three patient groups, while 20, 69, and 297 proteins were specific to NPDR, PDR, and NVG, respectively (Fig. 3*A* and supplemental Table S2). In the TMT platform, a total of 525 and 491 proteins were identified in the Set01 and Set02 technical replication experiments, respectively. To further explore the complementarity of the TMT and LFQ platforms, we compared the protein identifications at the gene level across both approaches. A total of 365 proteins were commonly identified between the TMT and LFQ datasets. Notably, 134 proteins were uniquely identified by the TMT platform, whereas 1365 proteins were uniquely identified by the LFQ platform (Fig. 3*A*). These findings underscore the impact of protein depletion protocols and workflows, with the LFQ platform's depletion strategy enhancing identifications, while the TMT platform's lack of depletion facilitates the detection of low-abundance proteins (supplemental Fig. S1) (22).

DEPs that met the stringent cutoff criteria (|Log_2_FC| >= 1 for LFQ, *p*-value <0.05 & |Log_2_FC| >= 0.58 for TMT) were considered as significant proteins in DR and further analyzed. Hierarchical clustering analysis, PCA, correlation analysis revealed that NPDR and PDR had more similar expression patterns compared to NVG (Fig. 3, *B*–*D*). Moreover, top upregulated proteins in each stage revealed a simple understanding of biological meaning of each stage. Specifically, in NPDR, CRYGC, CRYBB3, CRYGD, and CRYBB1 proteins, eye-structure proteins were relatively upregulated comparing to PDR. In PDR, HBD, HBB, HBA2, CA1, CAT, and BLVRB proteins, hydrogen peroxide catabolic process–related proteins were significantly enriched in PDR. Top NVG proteins are AHNAK, STC1, TF, SPINK5 and APOB, calcium transport–related proteins, and signaling receptor-binding proteins. A total of 313 DEPs between PDR and NPDR, 343 DEPs between NVG and PDR for LFQ, 48 DEPs between PDR and NPDR, and 68 DEPs between NVG and PDR for TMT were used for selecting final candidates for validation experiment, and finally common 49 proteins were selected as final candidates (supplemental Table S3).

### Functional Analysis of DEPs Revealed Three Significant Process in DR

**Fig. 4:**
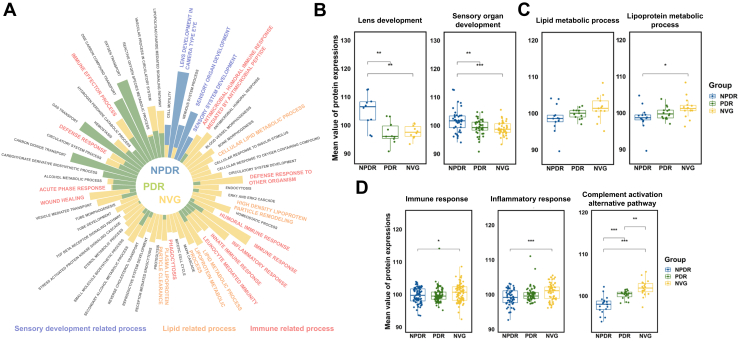
**Functional analysis using differentially expressed proteins.** Functional annotation analysis of ssGSEA. *A*, circular plot of significant biological process in DR. Bar plot are depicted using NES difference (each stage NES – mean value of every stage NES). Processes were validated with <0.05 FDR. *B*, protein box plot of significant process in NPDR. Lens development and sensory organ development were enriched. *C*, protein box plot of lipid-related process that shows gradual increase across stages. Lipid metabolic process and lipoprotein metabolic process were enriched. *D*, protein box plot of immune-related process. Immune response, inflammatory response, complement activation alternative pathway were enriched. Immune- and lipid-related process were enriched in NVG and PDR.

Gene set enrichment analysis with GO database were performed with entire quantifiable protein expression dataset in order to obtain a precise understanding of the mechanistic differences in DR (23). In the study, each stage of DR was analyzed using single-sample gene set enrichment analysis (ssGSEA). This method helped to pinpoint crucial processes by comparing the variations in normalized enrichment score and FDR values, as depicted in Figure 4*A*. Notably, processes of significance in NPDR were highlighted in blue. These included “sensory organ development” and “lens development.” In the case of PDR, key processes such as “oxygen transport” and “platelet aggregation” were notably enriched and indicated in green. For NVG, processes like “immune response” and “inflammatory response,” which play a critical role in the disease's pathogenesis, were denoted in yellow. A striking observation was the enrichment of lipid-related processes (“lipid metabolic process” and “lipoprotein metabolic process”) and immune-related processes (immune response, inflammatory response, and “defense response”) in both PDR and NVG stages. The ssGSEA analysis showed the involvement of three principal processes in DR: lens development, immune response, and lipid metabolic process. For a more precise understanding, we compared the alterations in protein expression pertinent to each process across different stages of DR, as shown in Figure 4, *B*–*D*. It was observed that the expression levels of proteins related to sensory organ development and lens development were markedly elevated in NPDR, but subsequently diminished in PDR and NVG (Fig. 4*B*). Proteins associated with lipid metabolism (lipoprotein metabolic process and lipid metabolic process) exhibited a progressive increase through the stages (Fig. 4*C*). Additionally, there was an ascending trend in the expression of proteins involved in immune responses, including immune response, inflammatory response, and “complement activation alternative pathway,” with a notable activation of the complement cascade (Fig. 4*D*). Particularly, most proteins in the complement cascade showed significant upregulation in the NVG stage (supplemental Fig. S2*A*). Network analysis further revealed that the intrinsic pathway of the coagulation cascade is specifically heightened in PDR and NVG. Processes like inflammation and vasodilation, driven by the Kallikrein-kinin system, and cell adhesion, migration, and proliferation, mediated by PLAUR, are predominantly active in PDR and NVG (supplemental Fig. S2*B*). Additionally, the “cytokine cytokine receptor interaction” is notably active in NVG. High expression levels of TNF family proteins, including FAS, TNFRSF1A, TNFSF12, TNFSF13B, TNFRSF12A, and TNFRSF21, were detected in NVG (supplemental Fig. S3*A*).

**Fig. 5:**
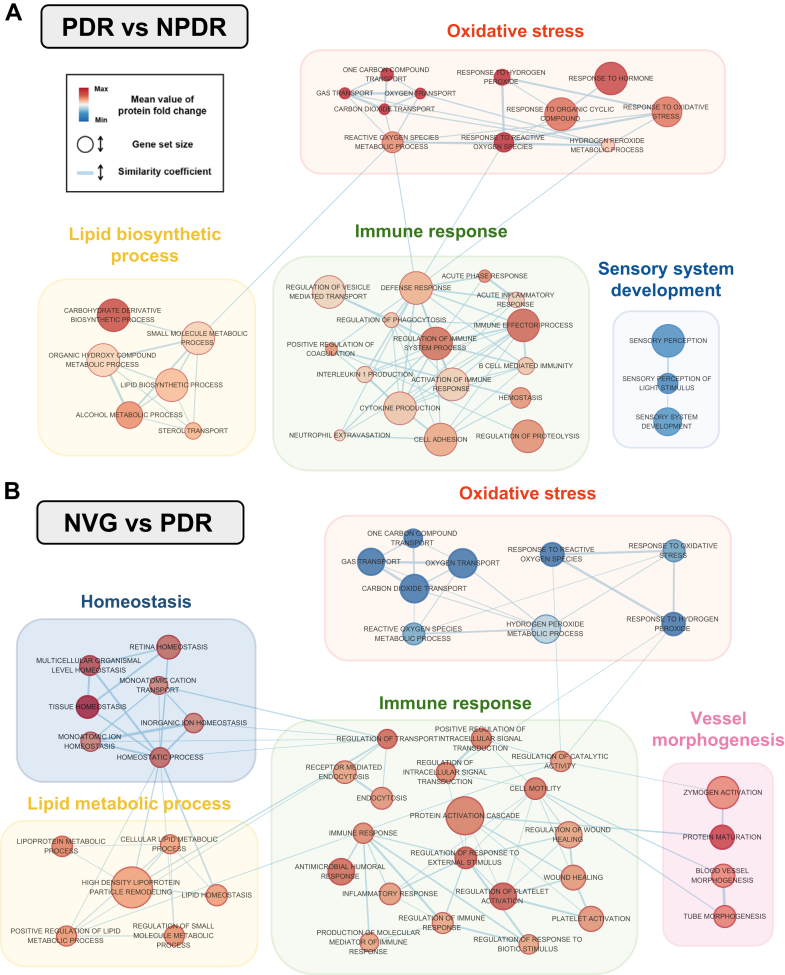
**Network modeling of AH proteins correlate with functional analysis.** Network was constructed using functional analysis results using enrichment map. The colors of the nodes represent the mean expression (fold change) of proteins involved in a gene set. Each gene set size means the number of proteins involved in a gene set. The connection between nodes (*blue* lines) shows similarity coefficient value. Thicker lines represent high similarity coefficient value. *A*, network model in PDR *versus* NPDR showing the biological processes, including immune response, Oxidative stress, sensory development process, and lipid biosynthetic process. *B*, network model in NVG *versus* PDR showing the biological processes including immune response, oxidative stress, lipid metabolic process, homeostasis, and vessel morphogenesis.

In Figure 5, network analysis utilizing the enrichment map method is employed to assimilate protein expression data, effectively mapping out the complex protein network involved in DR. This graphical representation reinforces the insights from the ssGSEA analysis presented in Figure 4. It shows a decrease in “sensory system development” during the PDR stage, suggesting early changes in neurosensory functions. Additionally, it highlights a progressive increase in lipid metabolic process and immune response as the disease advances from NPDR to PDR and further to NVG (Figs. 4*B* and 5*A*). The network also illuminates other pathways such as “oxidative stress,” “homeostasis,” and “vessel morphogenesis,” emphasizing their potential involvement in the pathogenesis of DR. Particularly, the pathways vessel morphogenesis and homeostasis are significantly more active in the NVG stage, a finding supported by Figure 5*B* and supplemental Fig. S3*B*. Furthermore, the analysis reveals a dual trend in oxidative stress: it initially rises as the condition progresses from NPDR to PDR and then diminishes from PDR to NVG (Fig. 5, *A* and *B*).

### Validation of Potential Biomarker Candidates Obtained From Profiling Experiment

The targeted proteomic approach in this study aligns with Tier 3 methods, employing targeted strategies to enhance the detection of a predefined set of analytes without constituting formal assays. Based on integrating proteomic results of LFQ and TMT and a literature review, we selected 49 proteins (711 transitions, supplemental Table S4) as potential candidate biomarkers. This transition list was optimized for dynamic MRM analysis, and consequently, we could narrow down 45 proteins (251 transitions with beta gal standard peptides, supplemental Table S4) that shows reproducible intensities for a single MRM run. After dynamic MRM analysis for optimized transitions, samples in which fewer than 50% of the total target transitions were detected were excluded (4 in NPDR, two in PDR). Additionally, the transitions exceeding 20% coefficient variance were excluded and only 44 transitions for 26 proteins were left for statistical analysis (supplemental Table S4). Finally, we could select 19 proteins (21 peptides satisfying area under curve >0.7, supplemental Table S5) which share similar expression pattern with profiling experiments. Additionally, proteins with altered expression at more advanced clinical stages were included as candidate biomarkers. The validation phase assessed the relative abundance of each candidate across the NPDR, PDR, and NVG patient groups, revealing 11 proteins with significant concentration changes (all adjusted *p* < 0.05) between NPDR and PDR groups, including CRYBA1 and CRYBB2. Between the PDR and NVG groups, 12 proteins, including APOB, CST6, CA1, CA2, and PLTP, showed statistically significant differences in concentrations (all adjusted *p* < 0.05; supplemental Table S5). Notably, seven proteins (CA1, CRYBB2, VWF, APOB, PLTP, SPINK5, and TF) were regulated correlating with DR progression and exhibited area under curves >0.9, indicative of their potential as biomarkers (supplemental Fig. S4).

The investigation utilized NMF clustering and an open target platform analysis based on profiling to establish the correlation between disease score and stage-specific proteins representing various stages of DR (supplemental Fig. S4 and supplemental Table S6). This comprehensive analysis identified a total of 26 stage-specific proteins, including CRYBB2 earmarked as NPDR-specific, associated with eye structure and development. This suggests alterations in eye structure proteins from NPDR to more advanced stages, as CRYBB2 demonstrated a high eye disease score, indicating its relevance to the stage. In the PDR stage, markers such as hemoglobin-related and structural proteins, along with CA1, known for its role in AH formation, exhibited high eye disease scores. The NVG stage revealed blood vessel development proteins, lipid localization proteins, and hydrolase activity–related proteins as specific markers. Among the seven markers from sequential proteomics analysis, CA1, VWF, PLTP, APOB, and TF, identified as PDR- and NVG-specific markers, demonstrated relatively higher diabetes mellitus scores than NPDR-specific proteins. Validated against eye disease and diabetes mellitus relevance, these stage-specific proteins underscore their significance in the progression of DR and highlight their potential as biomarkers for disease staging.

### Clinical Relevance of the Potential Biomarker Candidates

To assess the clinical relevance of the proteins identified in the validation phase, a regression analysis was performed using clinical variables such as age, cortical cataract, posterior cataract, ME, and PRP. This analysis revealed 24 peptides with significant adjusted *p*-values, providing insights into their associations with specific clinical conditions.

**Fig. 6:**
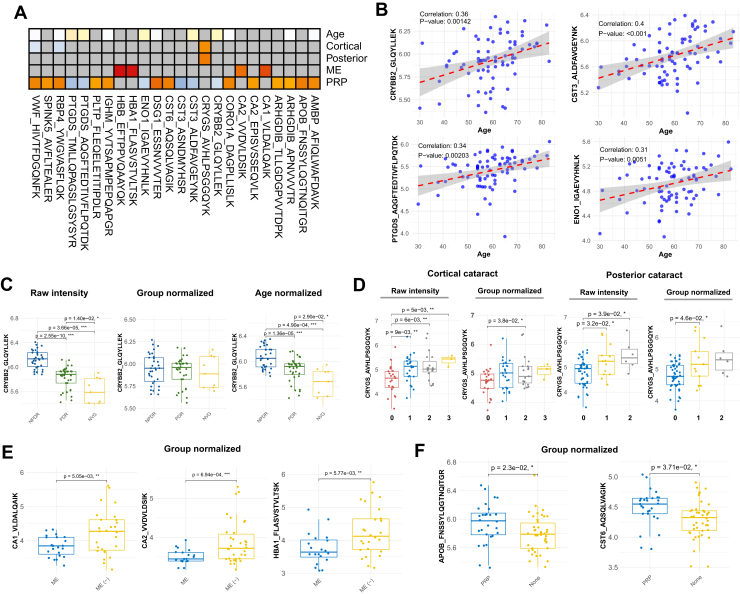
**Regression analysis and clinical correlation of key proteins with age, cataract grades, macular edema, and PRP treatment.***A*, heatmap displaying regression estimates of proteins significantly associated (adjusted *p*-value <0.05) with clinical variables, including age, cataract grades (cortical, posterior), macular edema (ME), and PRP treatment. The color scale represents regression estimates: *orange* indicates positive estimates, while *blue* indicates negative estimates, highlighting the direction and strength of associations. *B*, scatter plots showing correlations between protein intensities (*e.g.*, CRYBB2, CST3, PTGDS) and age, with significant associations observed despite minimal group effects. *C*, box plots of CRYBB2 intensities normalized by group and age, illustrating its association with age and diabetic retinopathy. *D*, box plots of CRYGS intensities normalized by group and cataract grades, illustrating its association with cortical and posterior cataract severity. *E*, box plots of CA1, CA2, and HBA1 intensities in macular edema, showing persistent significance after group normalization. *F*, box plots of APOB and CST6 intensities in PRP-treated *versus* untreated patients, highlighting their potential roles in therapeutic response.

Among these variables, age exhibited the lowest estimate values and achieved statistical significance. However, despite the significant *p*-values, age had minimal influence on group stratification. Even peptides strongly correlated with age, such as CRYBB2, CST3, PTGDS, and ENO1, maintained distinct differences between groups after age adjustment, suggesting that DR exerts a far greater influence on proteomic profiles than age (Fig. 6, *B* and *C*). To determine whether the proteomic differences observed in DR were influenced by age, we examined age-dependent protein expression patterns in the validation cohort, where PDR and NVG patients were generally younger, while NPDR patients were older, creating an age imbalance. Given this, we stratified patients based on the median age of 60 to evaluate whether proteomic differences persisted within age-matched subgroups. This showed that age-dependent proteins exhibited strong correlations with age in the ≥60 group but lost significance in the <60 group. Notably, CRYBB2 (GLQYLLEK) remained consistently upregulated in NPDR regardless of age, confirming its association with DR progression. In contrast, PTGDS (AQGFTEDTIVFLPQTDK), which had the highest age-related estimate, showed a slight reduction in statistical significance between DR groups but retained its overall expression pattern (supplemental Fig. S5). These findings suggest that while age influences some protein expressions, DR progression remains a primary factor in their regulation. Cortical and posterior cataracts, on the other hand, demonstrated a robust relationship with CRYGS, where the protein's expression remained significantly associated with cataract severity even after adjusting for group effects. This finding suggests that the increased aggregation propensity of CRYGS in more advanced cataract stages may drive these associations (Fig. 6*D*). To objectively assess whether cataract potentially influences DR, we performed an additional analysis to determine if the differences among DR groups persisted after adjusting for cataract severity. Patients were categorized by cortical cataract grades (0, 1, and 2), and age normalization was applied. This analysis revealed that CRYBB2 (GLQYLLEK) remained consistently elevated in NPDR across all cataract grades, indicating its independence from cataract status. In contrast, CRYGS (AVHLPSGGQYK) showed variability across cataract grades, with reduced DR group differences after cataract adjustment, suggesting an influence of cataract severity (supplemental Fig. S5). For ME, CA1, CA2, and HBA1 were identified as key proteins that retained significant associations with ME even after group adjustments. These proteins may reflect unique biochemical changes associated with ME and its influence on AH composition (Fig. 6*E*). Interestingly, PRP treatment was significantly associated with two proteins, APOB and CST6, even after accounting for group differences. The elevated levels of APOB likely reflect changes in lipid metabolism or inflammation induced by PRP, as APOB is a critical component of lipoproteins and has been linked to vascular remodeling. CST6, a cystatin protease inhibitor, may play a role in regulating extracellular matrix remodeling or protease activity following PRP treatment, potentially contributing to wound healing or tissue stabilization post-therapy. This observation highlights the importance of considering treatment history, such as PRP, in analyzing protein expression at diverse stages of DR. The interplay between PRP and secondary molecular changes, including those involving APOB and CST6, warrants further investigation to better understand their roles in treated *versus* untreated populations.

These findings collectively emphasize the significant impact of clinical variables on protein expression while also highlighting the overarching influence of DR pathology on proteomic profiles. They provide critical insights into the complex interplay between disease mechanisms, treatment effects, and clinical conditions in the context of DR progression and treatment.

## Discussion

Using MS-based proteomic analyses, our study characterized the AH proteome in patients with DR and its clinically important neovascular complications. The AH, composed of a complex mixture of proteins, growth factors, and electrolytes, plays a crucial role in maintaining a homeostatic microenvironment in the anterior segment structures of the eye. Abnormalities in the composition of AH can impact the surrounding intraocular tissues and contribute to ocular pathophysiology. Therefore, the AH proteome provides valuable insights into the molecular mechanisms underlying intraocular diseases.

In this study, we focused on exploring the differential expression of proteins among the NPDR, PDR, and NVG patient groups, with the aim of identifying biomarkers associated with the progression from NPDR to PDR and from PDR to NVG. The validation process confirmed the significance of the selected potential biomarker candidates obtained from the proteomic analysis. These candidates exhibited distinct expression patterns across different stages of DR, and their potential diagnostic value was supported by receiver operating characteristic curve analysis. Importantly, the stage-specific proteins identified through NMF clustering and open target analysis provided insights into the molecular changes associated with each stage of the disease and highlighted potential key proteins involved in the pathogenesis of serious neovascular complications of DR.

Particularly, our study contributes to the growing understanding of the molecular mechanisms underlying the progression of DR, with a specific focus on NVG. Recent studies on DR have underscored the importance of phenotyping to identify disease-specific biomarkers and therapeutic targets (24, 25). Likewise, advances in glaucoma research highlight the importance of patient stratification to develop tailored management strategies (26, 27), which are particularly valuable for severe subtypes such as NVG. By focusing on the unique molecular profile of PDR with and without NVG, we address an unmet clinical need, complementing existing studies while providing novel insights into potential biomarkers and therapeutic targets for NVG, which remains a particularly challenging condition to manage.

However, previous studies investigating the proteomic changes in patients with DR mainly used vitreous humor (VH) samples and focused on comparing DR to normal or diabetic control groups without DR (28, 29, 30). These studies identified a range of proteins associated with DR development, but few addressed the proteome associated with the complications of DR. Changes in protein concentrations within the AH may have significant effects on cellular function in the avascular tissues of the anterior chamber (31) and several studies suggested that the protein levels in the AH vary in different ocular disorders (32). As AH can be easily obtained by anterior paracentesis, AH proteome may provide a window to pathogenesis of intraocular diseases. The AH proteins may affect microenvironment of the iris and iridocorneal angle, the structures with pathologic neovascularization in NVG, which are far more important than vitreous proteins because AH is in direct contacts with the above structures. We therefore hypothesized that proteins mediating pathologic process leading to NVG may be better identified by proteomic analyses on AH samples than VH samples. Further, our study on AH proteome, by employing LFQ and TMT, included the greatest number of DEPs between DR and its complications. This comprehensive analysis allowed us to identify a larger pool of potential biomarker candidates for future therapeutic and diagnostic approaches to prevent blindness caused by the serious neovascular complications of DR.

Among the DEPs identified in our study, several candidates, such as APOB, TF, and S100A7, which are primarily involved in immune response processes, showed significantly different expression levels between NPDR and PDR, as well as between PDR and NVG. These proteins may serve as potential biomarkers for the transition from DR to more severe stages. Notably, crystallin proteins (CRYBA1, CRYBB2, CRYGS, and CRYGC) showed excellent performance in discriminating between NPDR and PDR, suggesting their potential as biomarkers specific to PDR. While the precise role of these proteins in the pathogenesis of DR requires further investigation, some of the DEPs have been previously associated with DR or laser therapy, which is a standard treatment for PDR and NVG.

Crystallin, traditionally recognized as structural components of the lens, have increasingly been implicated in retinal pathology and neurodegenerative conditions such as glaucoma. These proteins are expressed in the retina and play crucial roles in protecting retinal neurons from oxidative stress, inflammation, and apoptosis, processes that are central to the development and progression of DR (33, 34). Their dual functionality underscores their importance not only in lens development but also in maintaining retinal health in diabetic patients. In the context of our findings, the involvement of crystallin in both structural and neuroprotective roles suggest their potential contribution to the observed molecular changes in retinal and optic nerve pathology. This dual role highlights the complexity of crystallin function, warranting further investigation into their mechanisms and therapeutic potential in DR. However, it is important to interpret these findings with caution given the study's limitations, including the potential confounding effect of cataract. Further investigation with more rigorous control for cataract is warranted to clarify the mechanisms and therapeutic potential of crystallin in DR.

The enrichment analysis of DEPs revealed that the biological processes most affected in the transition from NPDR to PDR and from PDR to NVG were related to immune response. These findings are consistent with previous studies highlighting the role of inflammation in the pathogenesis of DR (35). Maladaptation of the innate immune system and dysregulated para-inflammation have been implicated in DR development and progression. Multiple inflammatory cytokines and chemokines have been found to increase in intraocular fluids of patients with DR. Local production of proinflammatory cytokines, rather than systemic production, appears to be more relevant to the pathogenesis of DR and PDR. In advanced stages of DR, the breakdown of the blood-retinal barrier may lead to the infiltration of circulating immune cells and serum proteins into the retina, contributing to chronic retinal inflammation and damage to retinal vasculature and neuronal tissues (35). While the association between immune response and advanced complications of DR has not been fully established, the dysregulation of immune-related DEPs in eyes with severe DR complications suggests that the regulation of pathologic inflammation in patients with NPDR may prevent serious complications and further vascular or neuronal damage. However, the pathogenic association between complications such as PDR and NVG and inflammation needs further investigation. It remains unclear whether these complications are triggered by inflammatory mediators or the leakage of blood proteins into the AH, which could be caused by the breakdown of the blood-ocular barrier and subsequent increase in inflammatory mediators in the AH. The identification of increased proteins as potential markers for serious complications of DR underscores the need for further investigation into the pathogenic association between these complications and inflammation.

Our study offers potential pathways for clinical implementation. The concept of an AH "liquid biopsy" has emerged as a promising approach for identifying biomarkers in patients with advanced complications of DR. Biomarkers associated with inflammation and angiogenesis have enhanced our understanding of DR pathogenesis and guided the development of novel therapeutic options. Recent drug development efforts focusing on inflammation in DR underscore the translational importance of these biomarkers. In this context, our study’s identification of specific proteins associated with the progression to severe DR complications contributes important insights to this evolving field. Additionally, the integration of proteomic profiling into clinical practice holds immense potential for personalized treatment algorithms. By tailoring therapy selection to an individual’s proteomic profile, treatment outcomes for DR patients can be optimized. Such approaches could enable more precise targeting of molecular pathways involved in DR progression, paving the way for personalized, pathway-targeted therapeutic strategies for DR patients. By addressing critical gaps in understanding the molecular mechanisms underlying these complications, our findings provide a foundation for future research aimed at improving clinical outcomes and guiding individualized patient care.

While our study provides valuable insights into the AH proteome and its association with the complications of DR, there are certain limitations that should be acknowledged. Firstly, the relatively small sample size of our study, particularly in the NVG group, may limit the generalizability of our findings. Therefore, future studies with larger NVG cohorts are needed to validate the potential associations observed and further explore the identified biomarkers. Future studies with larger cohorts are needed to validate and further explore the identified biomarkers. Additionally, the use of AH samples may not fully capture the molecular changes associated with DR and its complications. Further studies incorporating other sample types, such as VH or serum, would provide a more comprehensive understanding of the molecular alterations in DR. Finally, functional experiments are required to elucidate the precise roles of the identified biomarkers in the pathogenesis of DR and its complications.

Furthermore, distinguishing the effect of cataract from the severity or complications of DR was particularly challenging in our study. Ideally, we would have included NPDR, PDR, and NVG cases without cataract to exclude the effect of cataract; however, given that diabetes is a well-known risk factor for cataract, it was challenging to find cases in these groups without cataract. Moreover, although we considered controlling for cataract statistically, some patients had previously undergone cataract surgery, and preoperative cataract information was unavailable for some cases. Given these complexities, it is extremely challenging to fully control for the cataract variable, and we acknowledge that this limitation may introduce potential deviations in our results.

Another limitation of our study is the challenge of distinguishing primary pathogenetic factors from secondary proteomic changes resulting from significant structural and physiological alterations in advanced disease states such as PDR and NVG. Both conditions involve angiogenesis, breakdown of the blood-ocular barrier, and potential leakage of serum proteins into the AH, which can obscure the identification of proteins with direct pathogenic roles. Additionally, in NVG, elevated IOP introduces further complexity through cellular stress and tissue remodeling, significantly influencing the AH proteome. These secondary proteomic changes, driven by neovascularization-associated protein leakage, local ocular responses, and IOP elevation, may lead to the detection of nonpathogenic proteins associated with the transition from NPDR to PDR or NVG, complicating the interpretation of proteomic data.

Adding to these complexities, cataract status was not fully controlled, which may have introduced additional variability in our findings. Given that NPDR patients exhibited a higher prevalence of cataracts, certain protein expression changes could be influenced by cataract severity rather than DR progression itself. Notably, the DR biomarker candidates identified in this study exhibited consistent differences among DR groups within the cataract-controlled dataset, reinforcing their potential relevance to DR progression rather than cataract severity. Nevertheless, the limitations in cohort size and the inability to fully control for clinical variables remain significant constraints of this study. In particular, the small number of NVG samples in validation and the lack of standardization in cataract grading for a larger cohort may introduce variability in the analysis and affect the reliability of the findings. While the NVG-specific markers identified in this study show potential, they cannot yet be considered reliable biomarkers without further validation in larger cohorts. Therefore, future studies should incorporate larger, more rigorously controlled cohorts with standardized clinical parameters, including stratified patient selection based on treatment history, disease severity, and clinical characteristics such as age and cataract grade. This approach will be crucial in distinguishing primary disease drivers from secondary effects, ensuring that the identified proteomic changes truly reflect DR progression rather than unrelated ocular conditions while also refining these findings and establishing the clinical utility of the identified biomarkers.

In conclusion, our study characterized the AH proteome in patients with DR and associated neovascular complications. Through comprehensive proteomic analyses and validation processes, we identified potential biomarkers for the serious complications of DR. The stage-specific proteins identified in our study provide insights into the molecular changes associated with each stage of the disease and offer potential therapeutic targets for the prevention of serious neovascular complications and related blindness. Future research efforts should focus on validating and expanding upon our findings, as well as investigating the functional roles of the identified biomarkers in the pathogenesis of neovascular complications of DR.

## Data Availability

The MS proteomics data (DDA and MRM data) have been deposited to the ProteomeXchange Consortium (http://proteomecentral.proteomexchange.org) *via* the PRIDE partner repository with the data set identifier PXD058481.

## Supplemental data

This article contains supplemental data.

## Conflicts of interest

The authors declare that they have no conflicts of interests with the contents of this article.

## Figures and Tables

**Revised_Supplementary figure 1:**
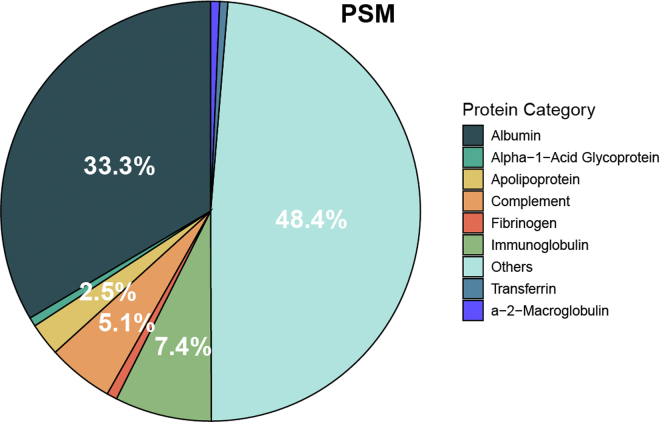


**Revised_Supplementary figure 2:**
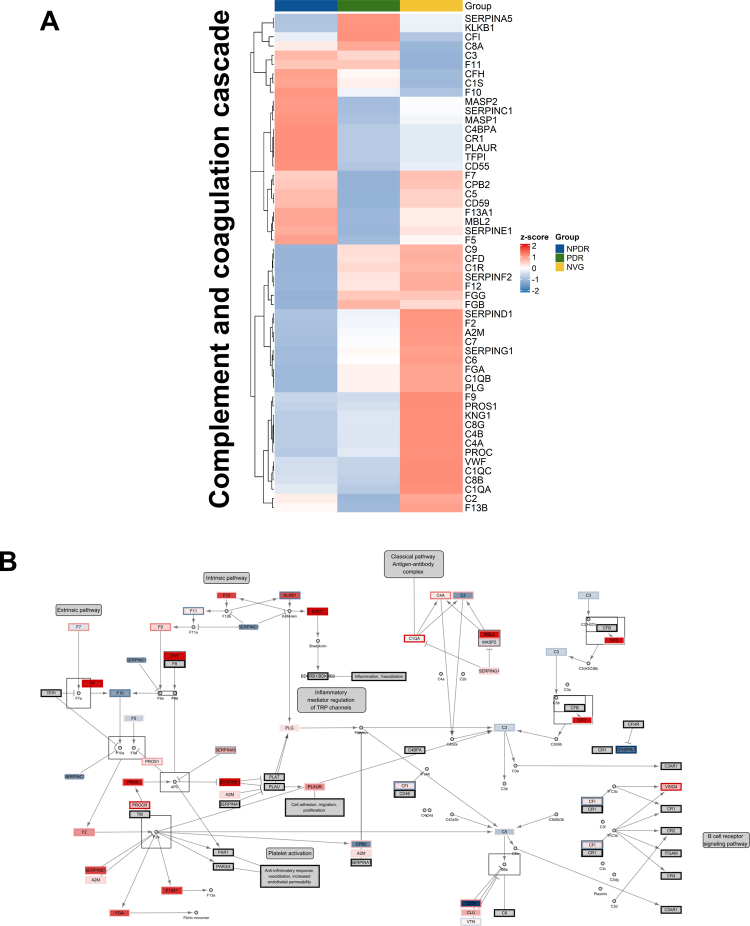


**Revised_Supplementary figure 3:**
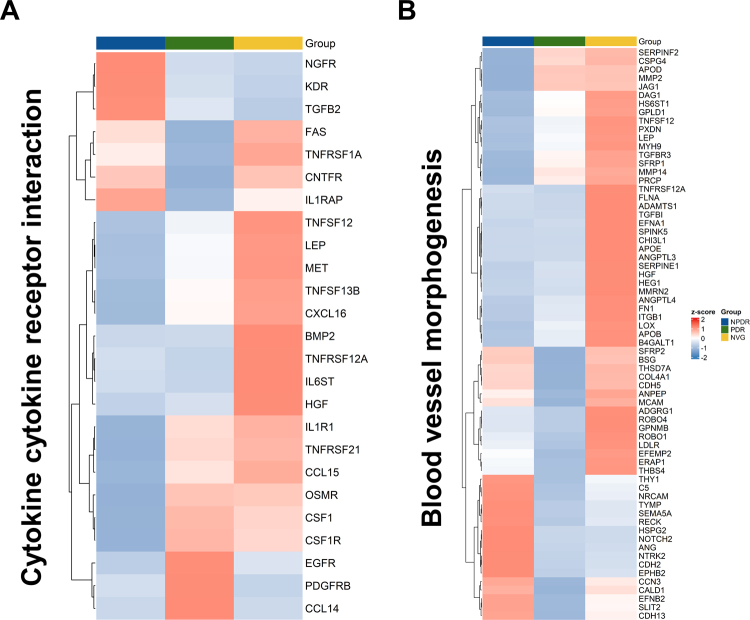


**Revised_Supplementary figure 4:**
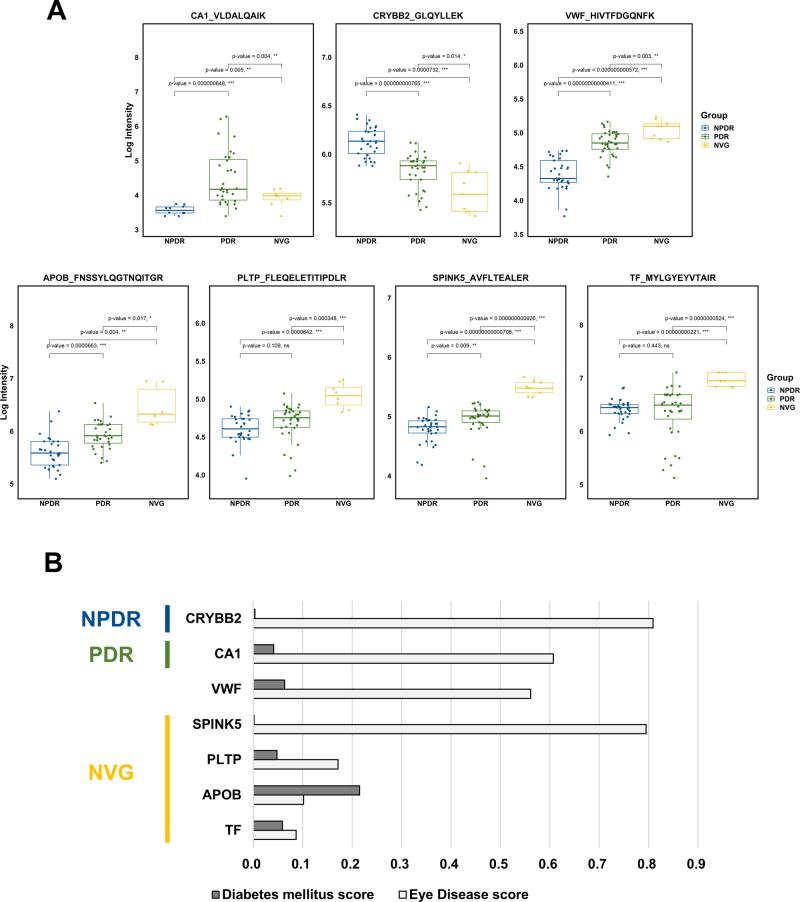


**Revised_Supplementary figure 5:**
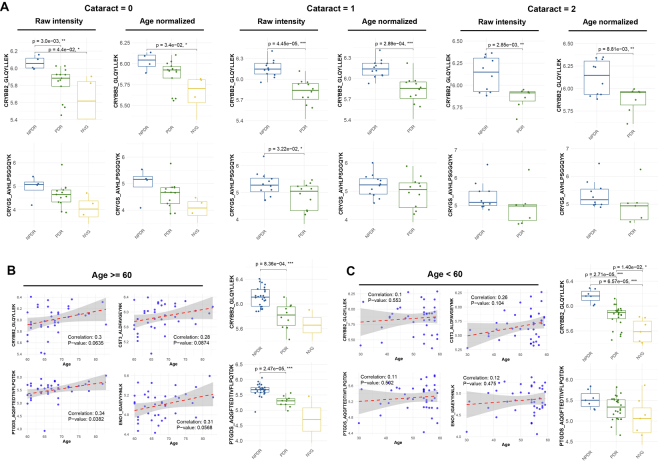


## Supplementary Data

Revised Supplementary Table 1

Revised Supplementary Table 2

Revised Supplementary Table 3

Revised Supplementary Table 4

Revised Supplementary Table 5

Revised Supplementary Table 6

Revised Supplementary file legends
