# Supplementary material for: Proteomic Analysis of Aqueous Humor Identified Clinically Relevant Molecular Targets for Neovascular Complications in Diabetic Retinopathy
Source: Mol Cell Proteomics. 2025 Mar 19;24(4):100953. doi: 10.1016/j.mcpro.2025.100953 (PMC12131854; doi:10.1016/j.mcpro.2025.100953)
Supplement: Revised Supplementary file legends [file mmc7.docx]

**Supplementary figures and table legends**

**Supplementary figure 1**

PSM Proportion of High-Abundant Proteins in Undepleted Aqueous Humor (AH) Samples
Pie chart illustrating the proportion of peptide-spectrum matches (PSMs) corresponding to highly abundant proteins in undepleted AH samples. The distribution highlights the dominance of specific proteins, reflecting the proteomic composition of AH before depletion. High-abundant proteins were identified based on their relative PSM counts, indicating their substantial contribution to the overall protein content in the sample. AH, aqueous humor; PSM, peptide-spectrum match.

**Supplementary figure 2**

A) Heatmap of ‘complement cascade’ related proteins in three stages of diabetic retinopathy. Scaled data from normalized maxLFQ intensity was used for generating heatmaps. As the disease processes, ‘complement cascade’ related proteins showed increasing expression pattern. B) Network of Complement and coagulation cascade.

**Supplementary figure 3**

A) DEPs in KEGG pathway (‘Cytokine-cytokine receptor interaction’) were visualized in heatmap. PDR and NVG have more up-regulated proteins of ‘Cytokine-cytokine receptor interaction’ rather than NPDR has.

B) DEPs in ‘Blood vessel morphogenesis’ were depicted in heatmap. Two comparisons (PDR vs NPDR, NVG vs PDR) DEPs are separated in order to visualize the difference clearly. Scaled value was used for constructing heatmaps. Generally, up-regulated proteins are mainly distributed in NVG.

**Supplementary figure 4**

Validation of biomarker candidates for diabetic retinopathy

(A) Box plot of DEP peptide expressions that satisfy > 0.9 AUC from MRM scan in three DR validation cohorts. (B) Relationship of novel biomarkers with eye disease and diabetes mellitus. Seven marker proteins which have more than 0.9 AUC value showed relatively high eye disease score calculated in ‘Open target platform’. This indicates that upper DEPs are already known as the markers for eye disease or have potential to be a selective marker for each stage in DR.; MRM, multiple reaction monitoring; AUC, area under curve

**Supplementary figure 5**

Effect of Standardized Clinical Information on Diabetic Retinopathy Groups
(A) Box plots displaying raw intensity and age-normalized protein expression levels in controlled cataract samples (Cortical Cataract = 0, 1, 2). CRYBB2 and CRYGS expression levels were used for visualization, with both raw intensity and age-normalized intensity represented. Statistical significance was assessed using appropriate tests, with p-values indicated. (B) Correlation analysis between age and age-dependent protein expression, along with box plots of age-dependent proteins in individuals aged ≥60 years. CRYBB2 and PTGDS were analyzed to assess the effects of age and diabetic retinopathy. (C) Correlation analysis between age and age-dependent protein expression in individuals aged <60 years. CRYBB2 and PTGDS intensity levels were used to evaluate the impact of age on protein expression.

**Supplementary Table 1**

Clinical information for profiling experiment and validation experiments.

1-1) Clinical information for profiling experiment

1-2) Clinical information for validation experiment

**Supplementary Table 2**

AH proteins identified among three diabetic retinopathy groups. (NPDR, PDR, NVG)

1-1) Protein identified using LFQ

1-2) Protein identified using TMT (SET01)

1-3) Protein identified using TMT (SET02)

**Supplementary Table 3**

3-1) DEPs in PDR vs NPDR. Comparing PDR and NPDR protein expression, DEPs were selected as differentially expressed proteins with strict cutoff (fold change >= 2 or < =0.5). 3-2) DEPs in NVG vs PDR. Comparing NVG and PDR protein expression, DEPs were selected as differentially expressed proteins with strict cutoff (fold change >= 2 or <= 0.5)

3-3) Differentially expressed proteins (TMT) (fold change >= 1.5 or <= 0.667 & p-value < 0.05)

3-4) Final list for validation experiments

**Supplementary Table 4**

Biomarker candidates transition list for optimizing dynamic MRM.

4-1) transition list for optimizing dynamic MRM.

4-2) optimized transition list for a single MRM run.

4-3) Transition list satisfying CV < 20%

**Supplementary Table 5**

Final biomarker candidates after profiling and validation experiments.

5-1) Similar expression patterns from both profiling and validation were considered for selecting biomarker candidates and only the peptides that satisfy more than 0.75 AUC were selected as final biomarker candidates. Eye disease score, Diabetes mellitus score from Open target platform were collected in order to understand the correlation between stage specific proteins from NMF clustering and eye disease.

5-2) Final DEPs relevant to clinical information. Regression analysis were held and estimate and p-value are calculated.

5-3) Clinical information dependent proteins

**Supplementary Table 6**

Stage specific proteins using NMF clustering. Feature score was calculated using NMF clustering rank algorithm.
